# Genomics clarifies taxonomic boundaries in a difficult species complex

**DOI:** 10.1371/journal.pone.0189417

**Published:** 2017-12-12

**Authors:** Jason Baumsteiger, Peter B. Moyle, Andres Aguilar, Sean M. O’Rourke, Michael R. Miller

**Affiliations:** 1 Center for Watershed Sciences, University of California, Davis, United States of America; 2 Department of Animal Sciences, University of California, Davis, United States of America; 3 Department of Wildlife, Fisheries, and Conservation Biology, University of California, Davis, United States of America; 4 Department of Biological Sciences, California State University, Los Angeles, United States of America; National Cheng Kung University, TAIWAN

## Abstract

Efforts to taxonomically delineate species are often confounded with conflicting information and subjective interpretation. Advances in genomic methods have resulted in a new approach to taxonomic identification that stands to greatly reduce much of this conflict. This approach is ideal for species complexes, where divergence times are recent (evolutionarily) and lineages less well defined. The California Roach/Hitch fish species complex is an excellent example, experiencing a convoluted geologic history, diverse habitats, conflicting species designations and potential admixture between species. Here we use this fish complex to illustrate how genomics can be used to better clarify and assign taxonomic categories. We performed restriction-site associated DNA (RAD) sequencing on 255 Roach and Hitch samples collected throughout California to discover and genotype thousands of single nucleotide polymorphism (SNPs). Data were then used in hierarchical principal component, admixture, and F_ST_ analyses to provide results that consistently resolved a number of ambiguities and provided novel insights across a range of taxonomic levels. At the highest level, our results show that the CA Roach/Hitch complex should be considered five species split into two genera (4 + 1) as opposed to two species from distinct genera (1 +1). Subsequent levels revealed multiple subspecies and distinct population segments within identified species. At the lowest level, our results indicate Roach from a large coastal river are not native but instead introduced from a nearby river. Overall, this study provides a clear demonstration of the power of genomic methods for informing taxonomy and serves as a model for future studies wishing to decipher difficult species questions. By allowing for systematic identification across multiple scales, taxonomic structure can then be tied to historical and contemporary ecological, geographic or anthropogenic factors.

## Introduction

There is still much we can improve about the taxonomic delineation of species. Although species delineation often seems arbitrary, it is nevertheless extremely important to categorize organisms in a way that allows for recognition, management and conservation of distinct groups as we move into the future. With climate change upon us, it has never been more important to quantify biological diversity [1–3] before it is irretrievably lost. Therefore, properly defining genera, species, subspecies, and population structure, both historically and contemporaneously, is a critical issue.

Contemporary genomic techniques are an excellent, if underutilized, tool for reconciling conflicts in taxonomy. Initially, these techniques were expensive to use and applicable to only a few economically important species. However, recent advances in next-generation sequencing have greatly lowered costs, allowing for exploration of lesser studied, non-model organisms [4]. Additionally, high resolution genomic datasets may allow for multiple questions to be addressed at one time and at different taxonomic levels, something often missing in previous genetic approaches (mtDNA, microsatellites, etc.).

One way genomic approaches can be useful to taxonomic investigations is their ability to resolve difficult species complexes. These groups reflect a complicated evolutionary history, with limited diagnostic morphological characters, widespread geographic locations, unclear connectivity and overall opaque hierarchical structure [5,6]. Contemporary conditions may also be masking differences between groups. This is especially true in freshwater systems, where differing selective pressures between systems may be less obvious and ancestral connectivity unclear across species ranges [7]. High resolution genomic datasets have the potential to reconcile these difficulties, allowing for a true understanding of the relationships within a given species complex.

The California (CA) Roach/Hitch complex is an example of a difficult species complex. California contains some of the most human modified ecosystems in the world, most invaded by non-native species [8,9]. Distributed throughout many of its watersheds are two endemic freshwater minnows (Cyprinidae), CA Roach (*Hesperoleucus symmetricus*) and Hitch (*Lavinia exilicauda*). Many questions have been raised concerning these species including whether they should be considered distinct genera, whether CA Roach is one or multiple species, if subspecies or distinct population segments exist within each species, and if populations result from introductions [10]. Earlier genetic studies [11,12] have attempted to clarify these discrepancies but lacked the power to be definitive and could not address all questions proposed.

Here we use the Roach/Hitch complex to illustrate how genomics can be used to clarify and assign taxonomic categories. We performed restriction-site associated DNA (RAD) sequencing on 255 Roach and Hitch samples collected throughout California to discover and genotype thousands of single nucleotide polymorphism (SNPs). These data were then used in hierarchical principal component, admixture, and F_ST_ analyses to resolve a number of ambiguities and provide novel insights across a range of taxonomic levels. For example, at the highest level, our results suggest the CA Roach/Hitch complex should be considered five species split into two genera (4 + 1) as opposed to two species from distinct genera (1 +1). At the lowest level, our results suggest Eel River Roach are not native but instead introduced from the nearby Russian River. Overall, this study provides a clear demonstration of the power of genomic methods, allowing for systematic identification across multiple scales that can then be tied to historical and contemporary ecological, geographic or anthropogenic factors which generated this taxonomic structure.

## Results

### RADseq provides high quality genomic resources

**Fig 1:**
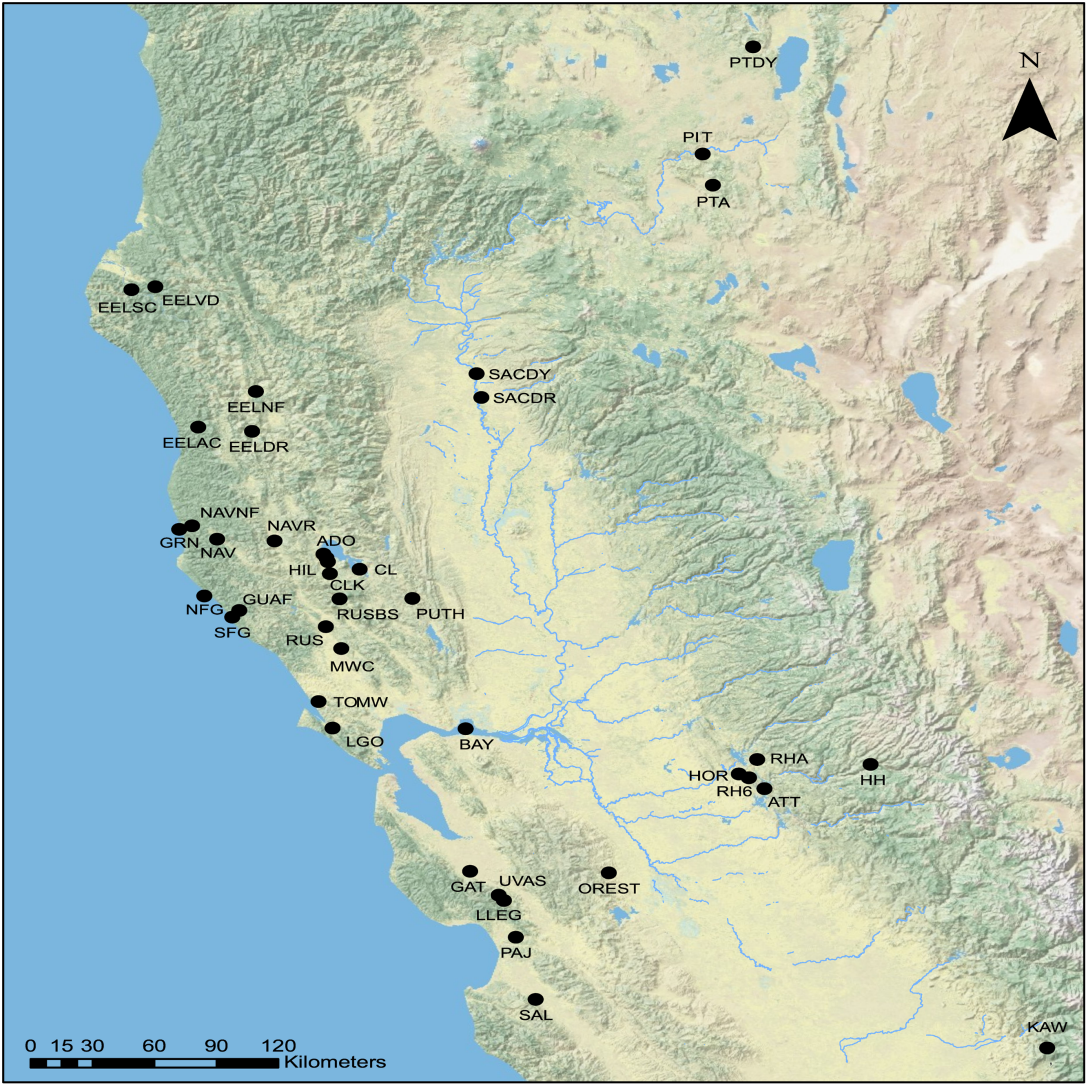
Topographical map showing 40 sampling locations throughout California. Full names and descriptions are available in S1 Table.

To begin investigating the CA Roach/Hitch species complex, we prepared restriction-site associated DNA (RAD) libraries by individually barcoding 280 samples (220 CA Roach and 60 Hitch) collected from 44 locations throughout CA (Fig 1; S1 Table) and sequenced them with paired-end Illumina technology. After sequencing, the barcode reads were partitioned by individual barcode and counted. Of the 60 Hitch individuals, 98% (59/60) had greater than 300k reads, with an average of 1.2m reads (Table 1). For CA Roach, 89% (196/220) of individuals had greater than 300k reads (ave. 1.3m). Overall, 91% (255/280) of individuals exceeded 300k reads, with an average of 1.25m. Given the genome size of these two species (approximately 1GB) and the restriction enzyme used (SbfI) in library preparation, 300k reads should provide a minimum of 5x coverage per locus [13], which is more than sufficient for population genomic analyses [14]. We conclude our data should be sufficient to facilitate robust analyses to discern hierarchical structure within the CA Roach/Hitch species complex.

**Table 1:** Sequencing information for the two *de novo* assemblies. Shown are the initial number of individuals, the average number of reads for those individuals, the number which exceeded 300k reads and the percentage of reads which aligned to each species partial reference genome (Ref).

	Reads			Alignments (%)	
	# samples	Ave	# > 300k	CA Roach Ref	Hitch Ref
CA Roach	220	1.3m	196	80	75
Hitch	60	1.2m	59	78	77

We next used the sequence data to generate two *de novo* partial reference (e.g. RAD locus) assemblies (one for CA Roach and the other for Hitch) because a pre-existing reference genome is not available for either species. To do this, we used a previously described pipeline [15,16] to discover and assemble loci with the paired-end sequencing reads. This process resulted in 22k loci with an average length of 471 bp for CA Roach and 19k loci with an average length of 437 bp for Hitch (Table 2). The total assembly size was 10.3 mbp for CA Roach and 8.7 mbp for Hitch, accounting for approximately 1.0% and 0.8% of their genomes. These assembly statistics were consistent with our expectations given genome size and restriction enzyme [13]. We conclude these *de novo* assemblies should be useful for interrogating genetic variation within the CA Roach/Hitch species complex.

**Table 2:** Partial genome statistics for the two species recognized at the onset of this study.

	Hitch	CA Roach
Number of Loci	19919	21834
Shortest length (bp)	300	300
Longest length (bp)	695	807
Ave. length (bp)	437	471
Total assembly size (bp)	8709173	10289652

To further investigate and compare the quality of these assemblies, we aligned the reads from all samples (both CA Roach and Hitch) to the Roach assembly and then repeated the alignments against the Hitch assembly. The roach samples had an average of 80% of reads aligned to the CA Roach reference whereas 75% of reads aligned to the Hitch reference. Similarly, for the Hitch samples, an average of 78% of reads aligned to the CA Roach reference and 77% of reads aligned to the Hitch reference (Table 1). Although similar, the alignment success was slightly higher with the Roach assembly for both species, suggesting the two genomes have similar content and low sequence divergence. This also suggested the CA Roach reference is slightly more complete and superior to the Hitch reference, although both reference assemblies produced identical biological results (see Methods; S2 Fig). We conclude that the CA Roach genome assembly is the better partial genome assembly for our study and it is consequently used throughout.

### CA Roach/Hitch complex consists of five species in two genera

The CA Roach/Hitch complex has a long history of ambiguous species designations. Snyder [17], using morphological characters, declared CA Roach individuals within the Central Valley along with those in the Navarro, Gualala, Russian, and Monterey Bay coastal regions to be distinct species. Furthermore, he posited that the most distinct Roach species was found in the Pit River (*H*. *mitrulus*—Northern Roach). An unpublished follow-up work by Murphy [18] classified all six of these as subspecies within a single CA Roach species *H*. *symmetricus*. This change was adopted by subsequent works [10,19–21] with no clear justification. As for genera, the American Fisheries Society (AFS) currently lists CA Roach (*Hesperoleucus symmetricus*) and Hitch (*Lavinia exilicauda*) as occurring in separate genera (e.g. [20]) based on Baird & Girard [22]. Schönhuth et al. [23] found some phylogenetic differences between genera at three nuclear loci but Moyle [10] and Aguilar & Jones [12] consider both species to be from a single genus (*Lavinia)* based on observed hybridization between species [24–26].

**Fig 2:**
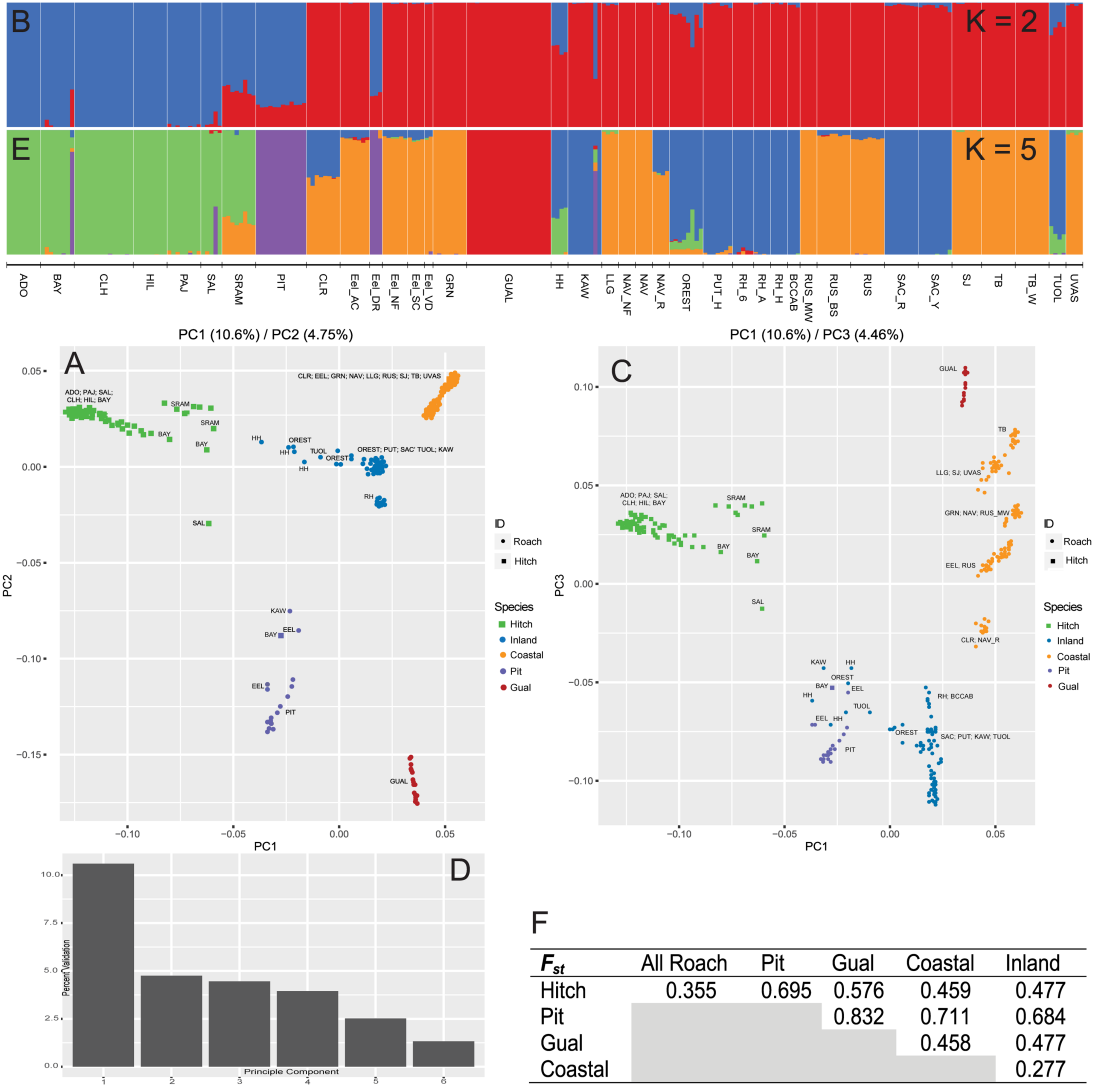
Summary genomic analyses for entire dataset. Shown is a principal component analysis for PC1 x PC2 (A) and PC1 x PC3 (C), with percent variation shown over six principal components (D). Admixture analyses on the same dataset were conducted for the two strongest supported clusters (K), two (B) and five (E), according to the Evanno method [27] shown in S1 Fig. Final pairwise estimates of F_ST_ between identified clusters (K = 5) are provided. All colors are consistent across subfigures, except (A) where blue represents *Lavinia* and red represents *Hesperoleucus*. Nomenclature is consistent with abbreviations in S1 Table.

To begin characterizing the genetic structure of this species complex, a conservative sample set (255 individuals with ≥ 300k reads) was aligned to the CA Roach genome and interrogated, revealing 690,046 raw single nucleotide polymorphisms (SNPs) (S2 Table). Called genotypes (with a minor allele frequency of 0.05 resulting in a more conservative 218,087 SNPs) were then used to generate covariates for principal component (PC) analyses. The first PC separated most individuals into two distinct groups (10.6% of the variation; Fig 2A) but a number of intermediates were observed, including all individuals from the Pit River basin. Admixture analyses (Fig 2B) supported a K of 2 after interrogating Ks of 1 through 8 (S1 Fig). The two clusters coincided well with the PC grouping. Individuals from some locations again showed intermediate admixture proportions; the most striking was from the Pit River basin. Here, all Pit River individuals assigned with approximately 80% ancestry to the cluster that contained primarily Hitch samples. This outcome was highly unexpected, given they are currently recognized as CA Roach.

To continue clarifying species boundaries within the complex, we examined additional principal components within the same 255 individuals. The second PC (4.8%) showed clear differentiation for Pit and Gualala River samples from all other CA Roach locations (Fig 2A). Additional groupings were also seen between geographically coastal and inland locations, a factor clearly defined by the third PC (4.46%) (Fig 2C). Further PCs analyzed showed similar patterns and decreasing information (Fig 2D). A continuation of the original admixture analysis also supported a K of 5 (S1 Fig) which coincided with clusters of Hitch samples, Pit River samples, Gualala River samples, samples from coastal watersheds, and samples from inland watersheds (Fig 2E), although a small number of individuals continued to show intermediate ancestry between clusters. These five clusters were further supported by F_ST_ analyses, with values ranging from 0.277 (Coastal vs. Inland) to 0.832 (Gualala vs. Pit) (Fig 2F). Applying different bioinformatic filtering criteria (see Methods) produced nearly identical results (S2 and S3 Figs). The consistency between independent analyses (PC and admixture) and strong genetic differentiation between each group/cluster clearly demarcated them as highly distinct genetic lineages. Unlike the original demarcation (K = 2), this secondary clustering of five (Pit, Gualala, Inland, Coastal, Hitch) does not ambiguously assign the Pit River samples, is consistent with previous studies, and makes the most biological sense. Therefore, if we take this genomic signal of K = 5 to be that of species designations, the best explanation for the original K = 2 and PC1 genomic signal is the delineation of genera. However results from all three analyses were not in strict agreement, preventing potential assignment of genera (see Discussion).

#### Clear Lake Roach have mixed Coastal and Inland ancestry

One location we anticipated might contain a cryptic species was the area surrounding Clear Lake, CA, where individuals do not reside within Clear Lake proper, only the tributaries immediately surrounding the lake. Most of the endemic fish species residing within or around Clear Lake have been identified as distinct species or subspecies [10]. CA Roach around Clear Lake are no exception, and were proposed as a subspecies in 1973 [18].

To investigate the potential distinctiveness of Clear Lake Roach, we examined the third PC (PC3—Fig 2C) from the above analysis. Although this demonstrated a clear distinction between geographically coastal and inland sampling locations, Clear Lake individuals appeared to be intermediate between the proposed Inland and Coastal species. Secondary analyses excluding Hitch, Pit and Gualala River samples; N = 156) found this same Coastal/Inland distinction on PC1 and all Clear Lake samples to be intermediate between proposed Coastal and Inland species (S4 Fig). Admixture analyses on the same secondary subset (N = 156) confirmed this assessment (K = 2; S1 Fig) and found Clear Lake individuals exhibited an approximately 60/40 Coastal/Inland ancestry (S4 Fig). Although it has been proposed that hybridization can lead to speciation [28], our data suggest Clear Lake individuals to be of mixed ancestry between proposed Coastal and Inland species as opposed to a distinct cluster (i.e. Clear Lake samples did not differentiate into a distinct cluster under higher K models as was the case with Pit River individuals). We also cannot rule out ongoing gene flow from the proposed Inland species. Finally, assignment of these individuals as a subspecies under one of the proposed species would be challenging, because no clear direction is evident. Therefore, a definitive conclusion regarding the taxonomic status of Clear Lake Roach cannot be made at present.

### Inland CA Roach exhibits a new subspecies (Red Hills Roach) and extensive population structure

Looking at taxonomic ambiguity within proposed Inland Roach requires focusing on the Great Central Valley of California. While highly distinct in its own right [29], within this region lies a unique ecological region known as the Red Hills, an Area of Critical Environmental Concern [30]. This area contains a number of endemic plants [25] and a morphologically distinct subspecies of “chisel-lip” Roach [31]. Subsequent genetic analyses have supported this claim [12,25] but were limited in their power to ascertain the taxonomy from a genetic perspective.

**Fig 3:**
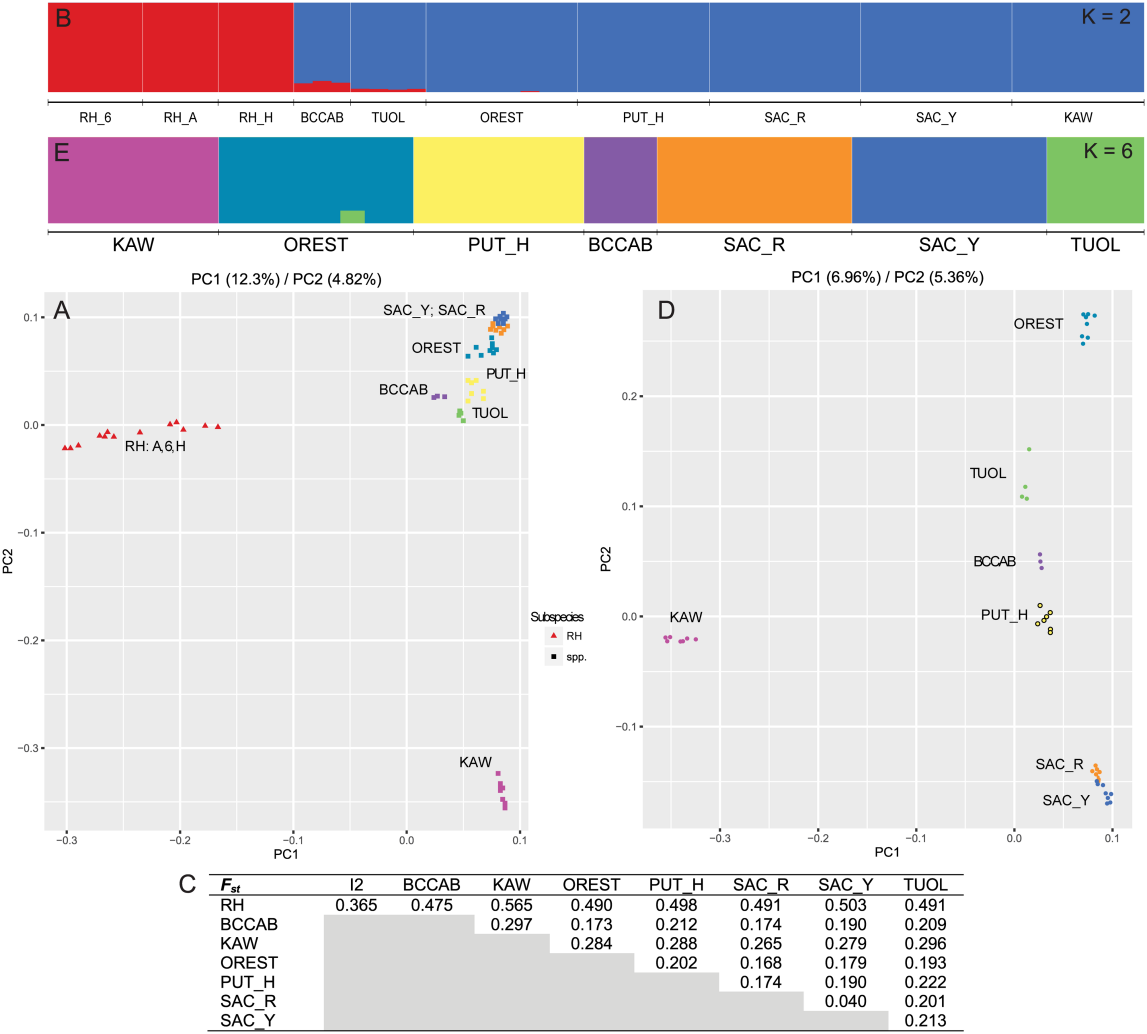
Subsample of data showing PC analysis, admixture and F_ST_ results for only individuals identified as the proposed Inland Roach species. Initial analyses (A–C) show results which include individuals sampled from the Red Hills region, a proposed subspecies of Inland Roach. Subsequent analyses (C–E) exclude these Red Hills individuals. Colors are consistent across all subfigures and nomenclature is consistent with abbreviations in S1 Table.

To ascertain any structure within the proposed Inland species, we restricted the dataset to 58 individuals from 10 locations around the Central Valley and reran all analyses under identical criteria as above. Principal component analyses strongly differentiated Red Hills locations (3) from all remaining locations within the Central Valley (12.3%; Fig 3A). Of the remaining locations, samples taken in the Kaweah River were distinct from all other locations on PC2 (4.8%), but some additional locational population structure was evident. Subsequent admixture analyses on the same dataset found a strongly supported K of 2 (S1 Fig); one cluster included all Red Hills locations and the second included all other locations (Fig 3B). Pairwise F_ST_ estimates reflected a similar pattern of substructure, with values ranging from (0.475–0.565) for any comparison between a Red Hills location and any other inland location (Fig 3C). A final pruning of the dataset to locations outside the Red Hills region found individual population structure in every sampled location (Fig 3D and 3E). Pairwise F_ST_ estimates were highest between Kaweah and any other location (0.265–0.296) and lowest between Sacramento River locations (0.040). The remaining comparisons fell between these extremes (Fig 3C). We propose that Red Hills Roach be considered a distinct subspecies within the proposed Inland Roach, with the Kaweah River serving as a distinct population segment. The remaining locations represent population structure within the proposed Inland species. Overall, the observed substructure continues to support our proposal that Inland Roach are a distinct species.

### Coastal CA Roach have two subspecies—Northern and Southern

**Fig 4:**
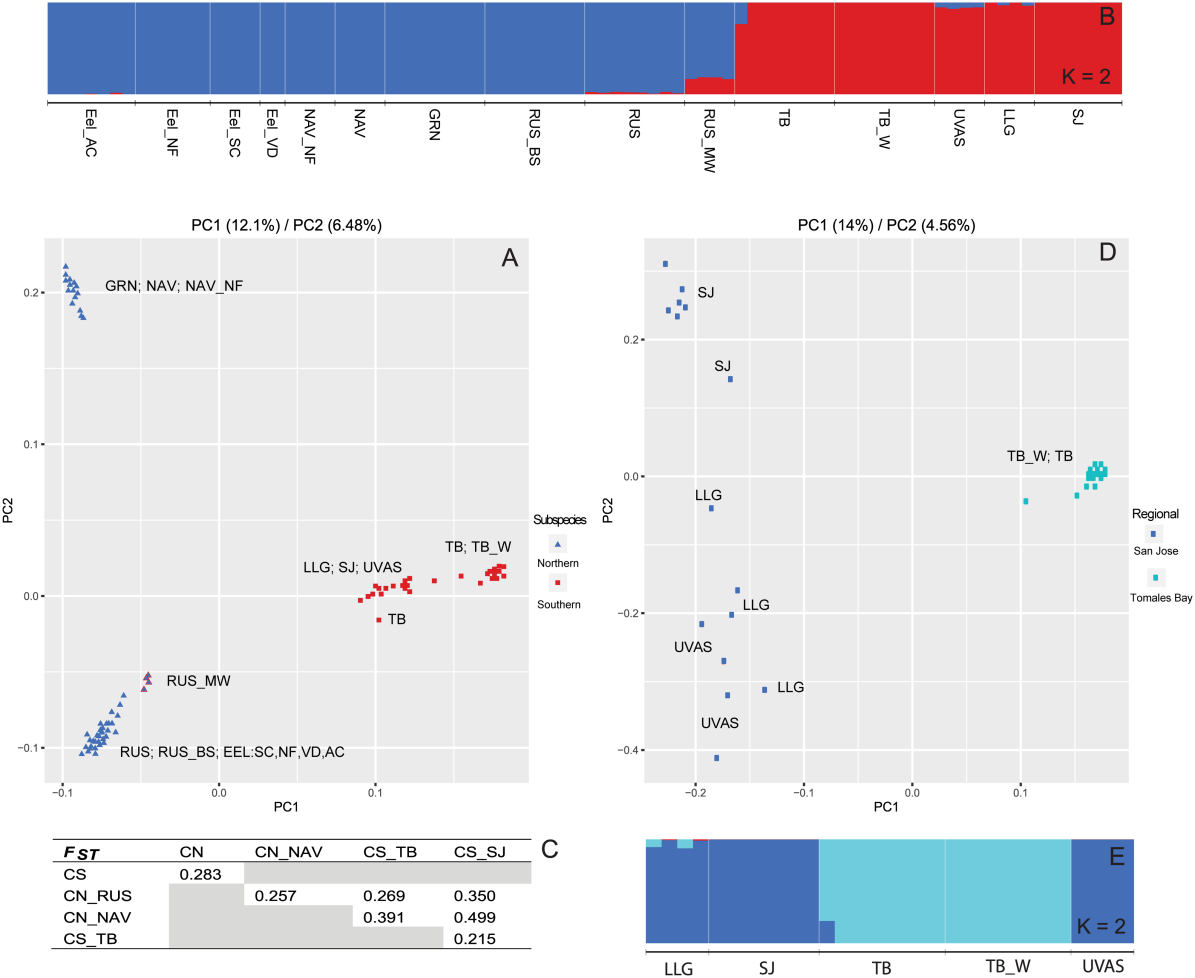
Subsample of data showing PC analysis, admixture and F_ST_ results for only individuals identified as the proposed Coastal Roach species. Initial analyses (A–C) show results which include individuals sampled from proposed Northern and Southern subspecies, along with any locations within these subspecies. Subsequent analyses (C–E) include only those locations from the proposed Southern subspecies. Colors are consistent between subfigures (A) and (B) and between subfigures (D) and (E) and nomenclature is consistent with abbreviations in S1 Table.

Looking at any taxonomic ambiguity within proposed Coastal Roach required focusing on all remaining coastal locations (excluding Gualala River), thus restricting the dataset to 15 locations (N = 86). Two additional locations were excluded: Navarro at Rancheria (as explained below), and Eel River at Dos Rios, which exhibited the same genetic signal as Northern Roach (Pit River) (see Fig 2E and Discussion). A PC analysis points to structure consistent with northern (Navarro-2, Greenwood-1, Eel-4, Russian-3) and southern (Tomales Bay-2, San Jose area-3) coastal locations (Fig 4A). Admixture analyses gave a K of 2 (S1 Fig), which is consistent with the same northern and southern groups outlined in the PC analysis (Fig 4B). The pairwise F_st_ estimate between northern and southern groups was 0.283. Fig 4C and finer-scale analyses (see below) revealed additional structure within each grouping, consistent with what one might see within a species/subspecies (Fig 4D and 4E). Therefore, we propose that Northern and Southern coastal groups be considered subspecies within the proposed species of Coastal Roach. Further, the observed substructure within Coastal Roach continues to support our claim of it being a distinct species.

#### Additional sub-structuring within each coastal subspecies

Addressing taxonomic structure within proposed Coastal subspecies meant reevaluating previous studies on coastal individuals. Historically, Roach from the Navarro River were identified as a distinct species [17]. Aguilar & Jones [12] also found unique genetic structuring for the Navarro and Tomales Bay locations, primarily with microsatellite data. A recent study on sympatric sculpin species in the San Jose and Russian River locations (Riffle Sculpin—*Cottus gulosus*) suggested locations contain a cryptic species of sculpin, vastly different from its inland counterparts [7].

**Fig 5:**
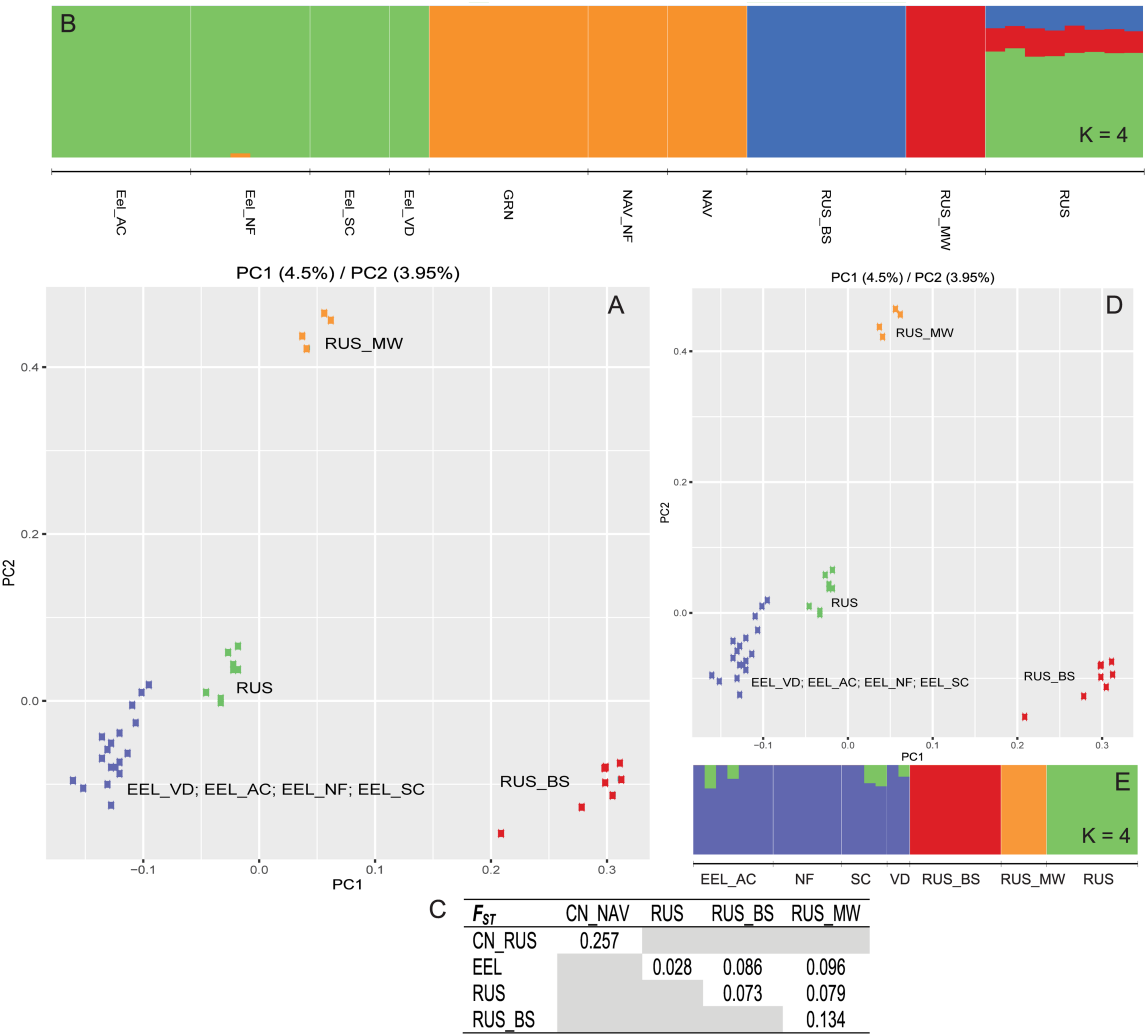
Subsample of data showing PC analysis, admixture and F_ST_ results for only individuals identified as the proposed Northern Coastal Roach subspecies. Initial analyses (A–C) show results which include a northern group (NAV/GRN) and a southern group (EEL/RUS). Subsequent analyses (C–E) include only those locations within the southern group and illustrate the population structure within the Russian River and absence of structure in the Eel River. Colors are consistent between subfigures and nomenclature consistent with abbreviations in S1 Table.

To address potential substructure within the southern subspecies of Coastal Roach, a finer parsing of data into only southern coastal locations (N = 31; 5 locations) found for both PC and admixture analyses a secondary grouping of Tomales Bay locations (2) and Monterey/San Jose locations (3) (Fig 4D and 4E). A similar sub-parsing for northern coastal locations (N = 55; 10 locations) found distinctive differences between Navarro/Greenwood (3) and Eel/Russian (7) locations (Fig 5A and 5B). F_ST_ estimates between each of these groups ranged from 0.215–0.499), with lower values for within subspecies estimates and higher values for between subspecies estimates (Fig 5C). Within subspecies F_ST_ estimates are consistent with those seen between other populations within this study (~0.200). We can draw the conclusion that differences seen in given systems in previous studies most likely represented subspecies differences as observed herein and that each system may only be a distinctive population within the proposed subspecies.

#### Individuals within the Eel River are introduced from the Russian River

The final ambiguity we wanted to address was the origin of Eel River Roach. Historically, CA Roach were thought absent from the Eel River basin despite its vast size and proximity to known CA Roach watersheds [10]. Yet relatively recent surveys have found CA Roach to be prominent in the Eel River [32,33], leading to speculation as to whether individuals were introduced from another watershed or whether early sampling efforts were insufficient to detect it.

To address the ambiguity, we used four well distributed locations within the Eel River (Del Rio was excluded—as stated above; see Discussion). Samples were compared to all Navarro, Greenwood, and Russian River locations using both PC and admixture analyses (Fig 5A and 5B). As previously observed, Navarro and Greenwood locations represented a single distinct population within the northern coastal subspecies. Further admixture runs (K = 3,4) did not show additional clustering except within the Russian River (S1 Fig). Removal of Navarro and Greenwood samples from the same analyses did not present any significant changes (Fig 5D and 5E). One location along the Russian River was noticeably closer to Eel River samples in the PC analysis (RUS) and the admixture analysis suggested shared ancestry between this site and a number of Eel River individuals. Pairwise F_ST_ estimates between Navarro/Greenwood and Eel/Russian groups were consistent with those seen between other populations of CA Roach in this study (0.257) (Fig 5C). A noticeable reduction in value was seen when Eel River samples were compared to any Russian River location, particularly RUS (0.028). If the Eel River had a historic population, we would expect to see distinctive structure within one or more analyses, sub-structuring within Eel River sampling locations similar to that seen in the Russian River and/or an F_ST_ estimate similar to other population-level comparisons in this study. The absence of all of these possibilities leads us to conclude that contemporary Eel River Roach are a relatively recent introduction from the Russian River, most likely from in or around the RUS sampling location.

### Limited structure within Hitch

There has been limited exploration of structure within the Hitch species. Currently three subspecies are recognized: Clear Lake (*L*. *e*. *chi*—Hopkirk [19], Monterey (*L*. *e*. *harengus*—Miller[34] and Sacramento (*L*. *e*. *exilicauda*—Murphy [17]. The most prominent is the Clear Lake subspecies, a lake-adapted form which has been listed as a threatened species [35]. Aguilar & Jones [12] found similar subspecies structure using mtDNA and microsatellites, but little to no population structure was evident within proposed subspecies.

Our sampling of this group was limited to 57 individuals from 7 locations (S1 Table). Unfortunately, none of those individuals or locations was from the Sacramento subspecies, making a more formal analysis incomplete. The most striking discovery came from individuals sampled in San Ramon, where clear admixture was observed with proposed Coastal Roach in both the PC (Fig 2A) and admixture analyses (Fig 2E). Similar analyses restricted to just Hitch were consistent with previous studies, showing two of the three proposed subspecies (S5 Fig). Interestingly, samples taken from San Francisco Bay streams clustered with Clear Lake in the admixture analysis, although PC2 showed a distinct grouping. However the proportion of variation explained on PC1 was relatively low (4.13%), as were F_ST_ values (Panel C in S5 Fig); in particular were those values between Clear Lake and Pajaro/Salinas River locations (0.072). If F_ST_ values are compared to those for other proposed subspecies within this study, they are substantially lower, similar with those between populations of CA Roach. Therefore, although results are fairly consistent with what is proposed for Hitch, a fine-scale analysis (which includes Sacramento fish) is needed to clarify whether these clusters represent subspecies or distinct population segments.

## Discussion

### An updated taxonomy for the CA Roach and Hitch complex

**Fig 6:**
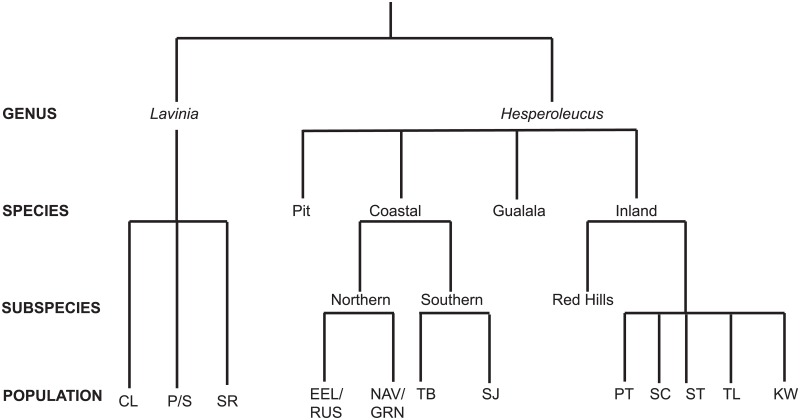
Proposed taxonomic hierarchy for the CA Roach and Hitch complex.

Rather than relying on previous information to make taxonomic designations, our study was run blindly and the structure ascertained solely on the combined genomic outcome of the three distinct analyses (PC, admixture, F_ST_) for identifying lineages. Strikingly, what resulted was a clear hierarchy, supported at each level by all three analyses (Fig 6). This hierarchy makes biological sense when considered with previous studies on these fishes, clearly defining lines obscured by multiple interpretations over time [12,17,18,24]. However, contemporary standards for delineating species have not adopted species identification based solely on genetic/genomic analyses [36]. Therefore, a second paper, which outlines all previous work, any morphometric/meristic distinctions, each species/subspecies range, the creation of type specimens and a formal nomenclature is required before “official” recognition as species/subspecies/DPS [37]. However, we look forward to a time when genomic techniques are included more prominently in identification of taxonomic lineages.

Two of the ‘levels’ of this hierarchy, we feel, warrant further discussion. The broadest and perhaps most difficult interpretation is which genomic signature represents the appropriate species delineation. Primary PC and admixture analyses show a clear demarcation for two groups, representing most contemporary Hitch and CA Roach, which could easily be interpreted as representing the two genera (*Lavini*a and *Hesperoleucus*) or two species (*L*. *exilicauda* and *H*. *symmetricus*). In theory, the strongest signal should be that of genera, the highest taxonomic level. Also, the first delineation (PC1 and K = 2) left Pit Roach in an ambiguous position, grouping more closely with Hitch than Roach. Furthermore, secondary assignment of five species strongly fits with previous descriptions [31]. Therefore we are inclined to conclude that the primary genomic signal, as demonstrated in both the admixture and PC analysis, is that of two genera.

The problem is the intermediacy of Pit River individuals and the assignment of their genus. Assignment to genus using our taxonomic approach should be straightforward, as admixture between genera is highly uncommon. But CA Roach appear to maintain relatively poor pre-zygotic barriers, as evidence from suspected hybridization with other more distantly related cyprinids such as Speckled Dace (*Rhinichthys osculus*) and Arroyo and Thicktail Chub (*Gila orcuttii* and *G*. *crassicauda*) [18,38]. In fact, hybridization appears to be common in much of Cyprinidae [39]. Thus, Pit River fish appear to be an example of hybrid speciation between ancestral Hitch and CA Roach. We recognize that hybrid speciation is not always well accepted [40,41] but it has been documented in fishes [42]. Additionally, admixture between other proposed species in this study (for example in Clear Lake) appears relatively common. Therefore the possibility of hybrid speciation is not without precedent.

Perhaps even more confounding is the admixture analysis assigned the majority of the genome (~80%) to Hitch but fish look morphologically like CA Roach [10]. With no clear pattern between analyses and marked complications in interpretation, we err on the side of caution and recommend Pit River individuals continue to be assigned to the genus *Hesperoleucus*. However, thanks to our genomic approach, a formal reevaluation can now be conducted to fully investigate how identified genomic differences define the contemporary species. What is not in dispute is that these individuals constitute a species at present, agreeing with Snyder’s original designation [17]. Future phylogenetic analyses may also help resolve problems but will likely struggle with the same mixed genomic signature. In the end, the power of genomic analyses for assessing taxonomy may reveal problems experienced here are more common than previously suspected, triggering a reevaluation of how organisms are taxonomically assigned.

The narrowest question proposed in this study was whether individuals within the Eel River represent a cryptic population (since there are no records prior to 1970 [32]) or whether they were introduced. Interestingly, a human-made tunnel (Potter Valley Project, 1908) diverts Eel River water into the East Fork of the Russian River and may confound interpretations [43]. Yet, subsequent taxonomic levels clustered Russian and Eel River samples together in every analysis. This is consistent with the dissertation of Jones [44], who proposed the Russian River to be the likely origin of Eel River fish (using mtDNA). Given the level of structure seen in proposed Coastal Roach in our study, it seems likely a system as large as the Eel River watershed [43] would represent a distinctive cluster if historically isolated. Furthermore, analyses restricted to Eel/Russian systems showed genetic structure for each sampling location within the Russian but none in the Eel. Again, if ancestral, we would expect substructure within the Eel similar to the Russian, a smaller overall system. Nevertheless, the Eel River could be identified as a distinct cluster in the admixture analysis with a severely pruned sample set and sufficient K. But this could be explained by the recent introduction of a limited number of individuals from the Russian River, followed by founder effects (essentially genetic drift) quickly altering the genetic makeup of Eel River individuals, leading to observed population structure [45]. And despite the connection between systems, the location within the Russian River most similar to contemporary Eel River fish was not from the East Fork, but further downstream. Therefore, the only sensible conclusion is the Eel River contains recently introduced Russian River fish. How these fish got into the Eel River remains a mystery (but see below).

### Anomalies in CA Roach/Hitch attributable to anthropogenic causes

The overall hierarchical structure identified within this species complex is consistent with a strong lack of gene flow likely brought on by geographic isolation at almost every location sampled. Structure is even observed between sites within a single system (e.g. Russian River—Fig 5), suggesting individuals are not moving over great distances. Yet despite these overwhelming patterns, a number of individual anomalies were detected. For example, one individual collected in San Francisco Bay as a Hitch actually clustered with Northern Roach (Pit River). Roach samples collected at Dos Rios along the Eel River also clustered with Northern Roach as well, despite the remaining four Eel River locations strongly clustering to proposed Coastal Roach (Fig 2E). Additional irregularities were seen for roach in the Navarro River (Rancheria Creek clustering with Clear Lake samples—S4 Fig). Potentially these anomalies represent errors in sample preparation or bioinformatic analysis, but stringent protocols were followed to ensure contamination or misidentification was avoided and we cannot find any evidence of sample swapping. Furthermore, samples for locations with anomalous individuals were collected on separate days and by separate individuals, making sample switching unlikely.

Our hypothesis is these individuals represent movement by humans as bait fish, a situation seen in multiple cases [46,47], because these fishes are small and hardy, so serve as excellent bait fish for introduced species like centrarchid basses. Once more, all sampled locations are easily within a day’s drive in California. Therefore, we believe these anomalies are the result of contemporary human movement as opposed to sample swapping in our analysis. And yet we do not think translocations are all that common, with most individuals dying from transport/treatment and survival highly contingent upon extenuating circumstances (like prolonged drought).

Perhaps the most perplexing of these anomalies comes from samples taken from Hetch Hetchy Reservoir. This reservoir, created by damming upper portions of the Tuolumne River in 1923, is a primary source of water for the city of San Francisco [48]. Individuals sampled here (and to a lesser degree downstream in the Tuolumne River) possess allele frequencies consistent with a mixture of Hitch and proposed Inland Roach (Fig 2E). A similar signal is seen between Hitch and proposed Coastal Roach in the San Ramon location (Fig 2E). Previous studies have shown that CA Roach and Hitch will hybridize when sympatric [23,24,34]. Therefore, it is likely these individuals have mixed ancestry. Hybridization in San Ramon could be explained by potential coexistence with Hitch but not Hetch Hetchy. To our knowledge, no Hitch are present in Hetch Hetchy Reservoir nor have they been introduced at any time by the California Department of Fish and Wildlife [10]. The only plausible explanation is introduction by unknown individuals, presumably as bait for the large brown trout (*Salmo trutta*) that inhabit the reservoir. However, we would be completely unaware of their mixed ancestry if it were not for the analyses performed here.

### The use of population genomics for assessing species complexes

When delineating species, it is important to have the right tools for the job. In the past, biologists often relied solely on morphological/meristic differences, which we now know can lead to erroneous conclusions [49]. For example, contemporary CA Roach were originally described using meristic and morphological traits from samples taken near Fresno, CA by Baird & Girard [21]. If a similar study were to be performed today (based on morphometrics/meristics), it is likely the anthropogenic movement of fish (identified in our study) would never be discovered. Instead, introduced variation in characters could lead to inconsistencies with the actual measures of the population, subspecies, or species. This would lead to improper metrics for identifying these lineages. Further, if locations were lumped (as is currently the case), these metrics would be further conflated, making it extremely difficult to assess differences. Finally, if ideas such as phenotypic plasticity [50] and crypsis [51] are introduced, the possibility of erroneous conclusions becomes substantial. Therefore, the importance of correctly identifying and preserving diversity is increasingly important, In the face of rapid change to ecosystems worldwide, we can ill afford to make mistakes which jeopardize our ability to manage and conserve lineages appropriately.

To combat some of these issues, biologists have employed genetic approaches (primarily traditional phylogenetic methods) to delineate species [52]. Phylogenetics can be highly effective for questions at the species level and above, comparing sequence differences at one or more loci, with the assumption that divergences at this scale (species and above) occur over comparatively longer periods of time [53]. Unfortunately, relying on a few genes (mtDNA for example) can be problematic, as phylogenetic trees represent the history of the gene(s) and not necessarily that of the species [54]. This problem is further compounded when species are relatively young (evolutionarily speaking). Many of the lineages may still be incipient species or subspecies, without sufficient time to be fully distinct. Problems with incomplete lineage sorting and admixture may also be rampant, obscuring the true history of the lineage in question [55,56]. This is exactly what is happening in a species complex such as the one investigated here.

Given these concerns, taxonomists can learn from the basic approach of population genetics to find the best way to quantify species complexes. After all, what is a species other than a distinct, self-sustaining population which has persisted over a long period of time? The many difficulties faced by population geneticists in assigning population structure (such as incomplete lineage sorting, admixture, migration, etc.) have been incorporated into its approach, which provides a more effective assessment of everything from classic population structure to relationships among true species [57]. Therefore, we would argue that population genetic approaches are better suited than standard taxonomic approaches, for delineating species and/or the hierarchy of structure within a particular species complex.

This population genetic approach is further improved with the addition of genomic methods, looking at thousands of loci rather than just a few. The statistical power associated with thousands of loci makes population genomic methods vastly superior to earlier techniques [13]. Gone are problems associated with limited loci or markers like microsatellites which require user interpretation [58]. Instead, results are more repeatable and with the amount of data available, questionable loci can simply be removed, providing a still robust way to look at variation across the whole genome of the organism. This abundance of variation also allows for fine-scale analyses, picking up the slightest variation between lineages. This is exactly what is needed for ascertaining structure of lineages within a species complex and, perhaps more importantly, for delineating species.

### A genomic approach to taxonomy

We have developed a relatively straightforward genomic framework that allows for improved assessment of structure and delineation of species, particularly within a species complex. Rather than restrict potential outcomes with prior information, we allowed the genomic information to dictate results, giving us a “natural” hierarchy within the individuals sampled. As a result, little human interpretation is necessary, making species delineations more consistent and defensible, because what constitutes a species is often debated [59,60]. Interpretations are instead limited to how or why lineages have attained this structure, an open and exciting field of study not directly addressed by our genomic analyses. Biologists are instead encouraged to apply applicable information in ecology, evolution, geography, geology, and other fields to develop their best theoretical hypotheses for the origins of identified lineages. Interestingly, this approach often represents the opposite of how biologists tend to approach such problems currently. We propose our genomic approach should precede most studies on how and why divergence occurred going forward. Once species, subspecies, and/or population structure is confirmed or reconfirmed, then the how and why can be most appropriately investigated. Otherwise, inferences are made without all the information available, a factor clearly seen in our study (unforeseen cryptic species, hybridization, and potential human movement of fish).

It has never been more important to properly designate species. With extinction levels rising due to anthropogenic causes [3,61], we need to quantify and protect our remaining diversity before it is gone. For most countries, this legal protection starts at the species level [62,63]. Therefore it is imperative that a consistent and reasonable interpretation of what constitutes a species should be implemented. Population genomics, as applied here, is a powerful method for delineating species-level structure and below (subspecies, distinct population segments, etc.). The sooner this approach is widely used, the sooner we can optimize management and conservation of much of the remaining diversity on this planet.

## Methods

### Sampling and DNA extraction

The original sampling was done by [12] and supplemented with additional samples collected over two years (2015–2016). Samples were collected based on the metrics of each species found in Moyle [10]. Locational information, including numbers obtained, is shown in Fig 1 or available in S1 Table. Fin clips were taken from live, unanaesthetized adults obtained via Smith-Root backpack electrofisher and handheld nets, dried on Whatman qualitative filter paper (Grade 1), and stored at room temperature. Clipped individuals were allowed to recover in a second bucket for a minimum of ten minutes prior to release. DNA was extracted with either the DNeasy Blood & Tissue Kit (Qiagen) or a magnetic bead-based protocol [64] and quantified using Quant-iT PicoGreen dsDNA Reagent (Thermo Fisher Scientific) with an FLx800 Fluorescence Reader (BioTek Instruments).

### RADseq

SbfI RAD libraries were prepared with well and plate barcodes using the RAD protocol of Ali et al. [64] and sequenced with paired-end 100 bp reads on an Illumina Hiseq 2500. RAD sequencing data was obtained by requiring an exact barcode and partial restriction site match [64]. A *de novo* assembly of a partial reference genome was assembled from the data using six Pit River individuals with an above average number of reads. A bioinformatic pipeline [15] in combination with a genome assembler (price—[65]), was used to construct a partial reference for each species (CA Roach and Hitch). No locus within the partial genome was included if less than 300 bp in length as the vast majority of the paired end data produced loci at least this length. Most, however, were much larger (ave ~450 bp—see Table 2).

Sequences were aligned to each reference assembly using the backtrack algorithm of bwa under default parameters [66]. samtools [67] was used to sort, filter for proper pairs, remove PCR duplicates, and index BAM files. Additional BAM file sets were generated to account for technical variation among individuals. To remove variation associated with variable sequencing depth, a set of subsampled BAM files were generated using samtools to randomly sample approximately 100,000 alignments from paired-end BAM files. This allowed for a normalization of the read number of each individual.

### Population genomic analyses

All RAD analyses were performed using angsd [68] with a minimum mapping quality score (minMapQ) of 10, a minimum base quality score (minQ) of 20, and the samtools genotype likelihood model (GL 1; [69]). Unless otherwise noted, samples with fewer alignments than required for subsampling were excluded and only sequence sites represented in at least 50% of the included samples (minInd) were used. These criteria, and each subsequent genomic analysis below, were repeated at each level of the hierarchical subsampling. Because the above approach is focused solely on variable SNPs, the number of loci at each hierarchical level slowly decreases, as some loci no longer contain informative sites for that particular subset of individuals. However because the initial dataset is so robust, reductions in informative SNPs (and therefore loci- see S2 Table) are minimal, as expected, and do not interfere with overall results (see QA/QC below).

Principal component (PC) analyses were performed by identifying polymorphic sites (SNP_pval 1e-6), inferring major and minor alleles (doMajorMinor 1; [70]), estimating allele frequencies (doMaf 2; [71]), and retaining SNPs with a minor allele frequency of at least 0.05 (minMaf). The number of SNPs used at each hierarchical level is available in the supplementary section (S2 Table). PC analyses were conducted on subsampled BAM files using genotype posterior probabilities calculated with a uniform prior (doPost 2). The ngscovar [72] function implemented in ngstools [73] was used to calculate a covariance matrix from called genotypes. The matrix was then plotted in R [74].

Admixture analyses were conducted on samples (based on each hierarchical subsampling) used in the PC analyses. To assuage concerns over linked SNPs due to the assumption of independence between sites, all loci for the admixture analyses were reduced to one SNP per locus based on the SNP with the highest minor allele frequency. These modified Beagle files [75] were used in ngsadmix [70]. The number of clusters (K) interrogated were adjusted according to each initial BAM file. Final K determination was conducted following the principles of [26], although other Ks were observed for additional potential information. Visualization was performed in R or with pophelper [76].

Genome-wide F_ST_ between population pairs (based on each hierarchical subsampling) was estimated by first estimating a site frequency spectrum (SFS) for each population (doSaf; [77]) using paired-end BAM files. Two-dimensional SFS and global F_ST_ (weighted) between each population pair were then estimated using realSFS [68].

To calculate Watterson’s theta [78] and Tajima’s theta [79], SFS estimated as described above were used as priors (pest) with paired-end BAM files to calculate each statistic for each site (doThetas). Sites were then averaged to obtain a single value for each statistic for each cluster identified in this study (and subsequent BAM file) [80]. Results showed little to no variation between values and are summarized in S3 Table.

### QA/QC

For any bioinformatic genomic project, the application of different filtering criteria at different times can influence downstream analyses. Although we used minimum filtering, some discrepancies may exist. To address this point and demonstrate the robustness of the data, we adjusted different filtering criteria and reapplied the same Admixture analyses as above. The minimum number of individuals (minInd) was adjusted to require only loci found in 50, 80 and 95% of individuals and a different *de novo* set of individuals (Hitch) was used, also adjusted for minInd (S2 Fig). To demonstrate that linked loci have no effect on the Admixture results in this study (even though we ran 1 SNP per locus), we ran each hierarchical analysis on the full set of available SNPs at that level. Results were identical, demonstrating our dataset is robust to assumptions of independence in these types of analyses and that cutting data down to a single SNP per locus was not necessary (S6 Fig).

## Supporting information

S1 Fig Graphical representation of clusters (K) supported by Evanno et al. (2005) for different hierarchical levels within the study. (PDF)

S2 Fig Repeated admixture analyses showing K = 5 based on different bioinformatic filtering. Loci present in at least 50%, 80% and 95% of individuals (minInd) based on original Pit Roach *de novo* assembly and a second filtering (50%, 80%, 95%) for a *de novo* assembly based on Hitch.
(PDF)

S3 Fig Graphical representation of original admixture analysis (K = 5) based on all loci (using a single highest minor allele frequency SNP per locus) and a second series of five random draws of 1000 loci where only 1 SNP was present per locus. (PDF)

S4 Fig Subsample of data showing PCA and admixture results for individuals identified as proposed Inland Roach and proposed Coastal Roach. Analyses show individuals from the Clear Lake region and upper portion of the Navarro River (Rancheria) appear to be intermediate between proposed species. Colors are consistent across subfigures and nomenclature is consistent with abbreviations in S1 Table.
(PDF)

S5 Fig Subsample of data showing PCA, admixture, and F_ST_ results for only individuals identified as the true Hitch species. Substructure within samples shows three distinct populations, supported by F_ST_ values consistent with population structure seen throughout the study. Colors are consistent across subfigures and nomenclature is consistent with abbreviations in S1 Table.
(PDF)

S6 Fig A reanalysis of each hierarchical level of admixture analysis using all possible SNPs per locus. Colors are unique to each figure and do not correlate between figures. Results are identical to one SNP per locus used in paper.
(PDF)

S1 Table Basic geographic information regarding sampling sites for CA Roach and Hitch. (DOCX)

S2 Table Overall single nucleotide polymorphism (SNP) counts at each level of the taxonomic hierarchy. All values were obtained with a minor allele frequency of 0.05, except those denoted by an asterisk. Clear Lake samples are not represented after inclusion at the All Samples and All Roach levels.
(DOCX)

S3 Table Watterson’s (Tw) and Tajima’s (Tp) theta (Ɵ per site) for each cluster identified. (DOCX)
