# Supplementary figures and images for: Genomics clarifies taxonomic boundaries in a difficult species complex
Source: PLoS One. 2017 Dec 12;12(12):e0189417. doi: 10.1371/journal.pone.0189417 (PMC5726641; doi:10.1371/journal.pone.0189417)

**All Samples**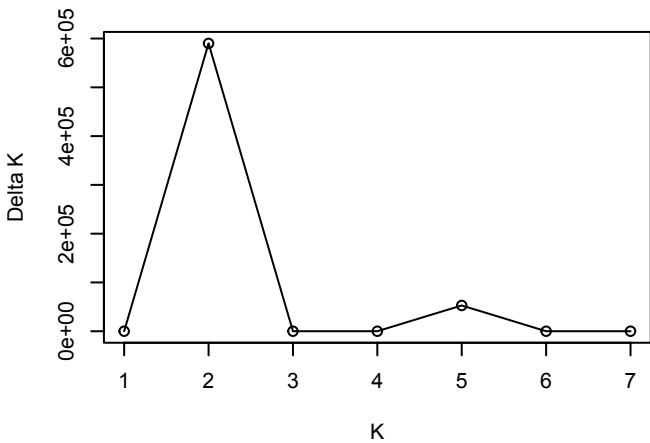**All Coastal/Inland Roach**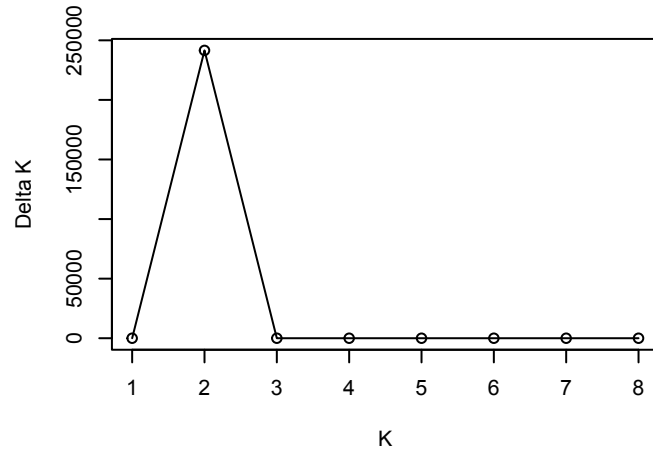**Hitch**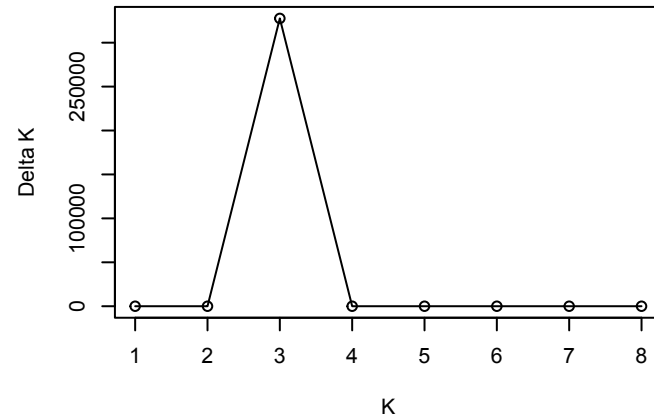**Pit Roach**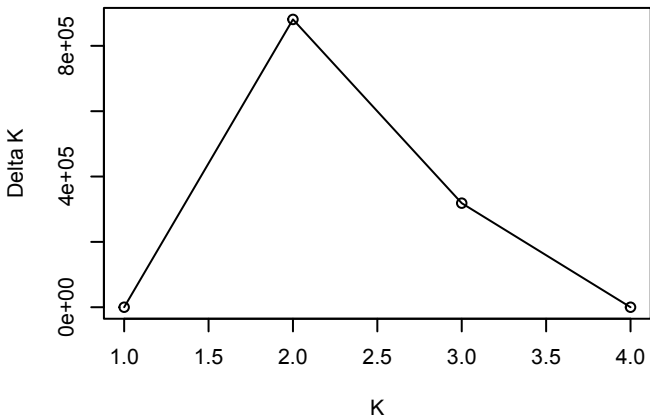**Inland Roach**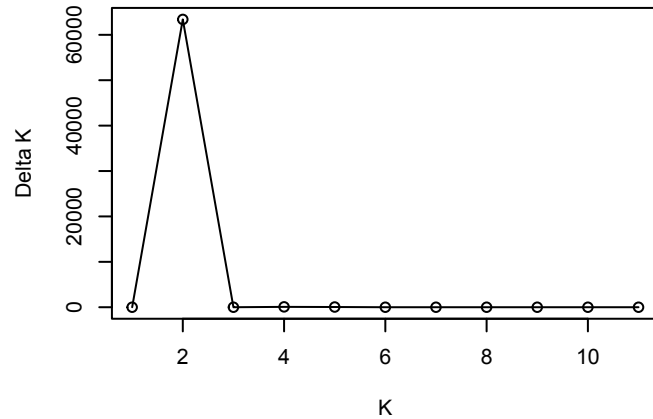**Coastal Roach**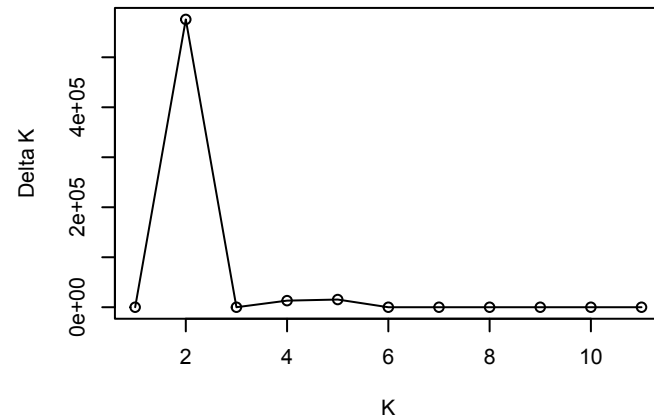

Supplement: S1 Fig — (PDF) [file pone.0189417.s001.pdf]

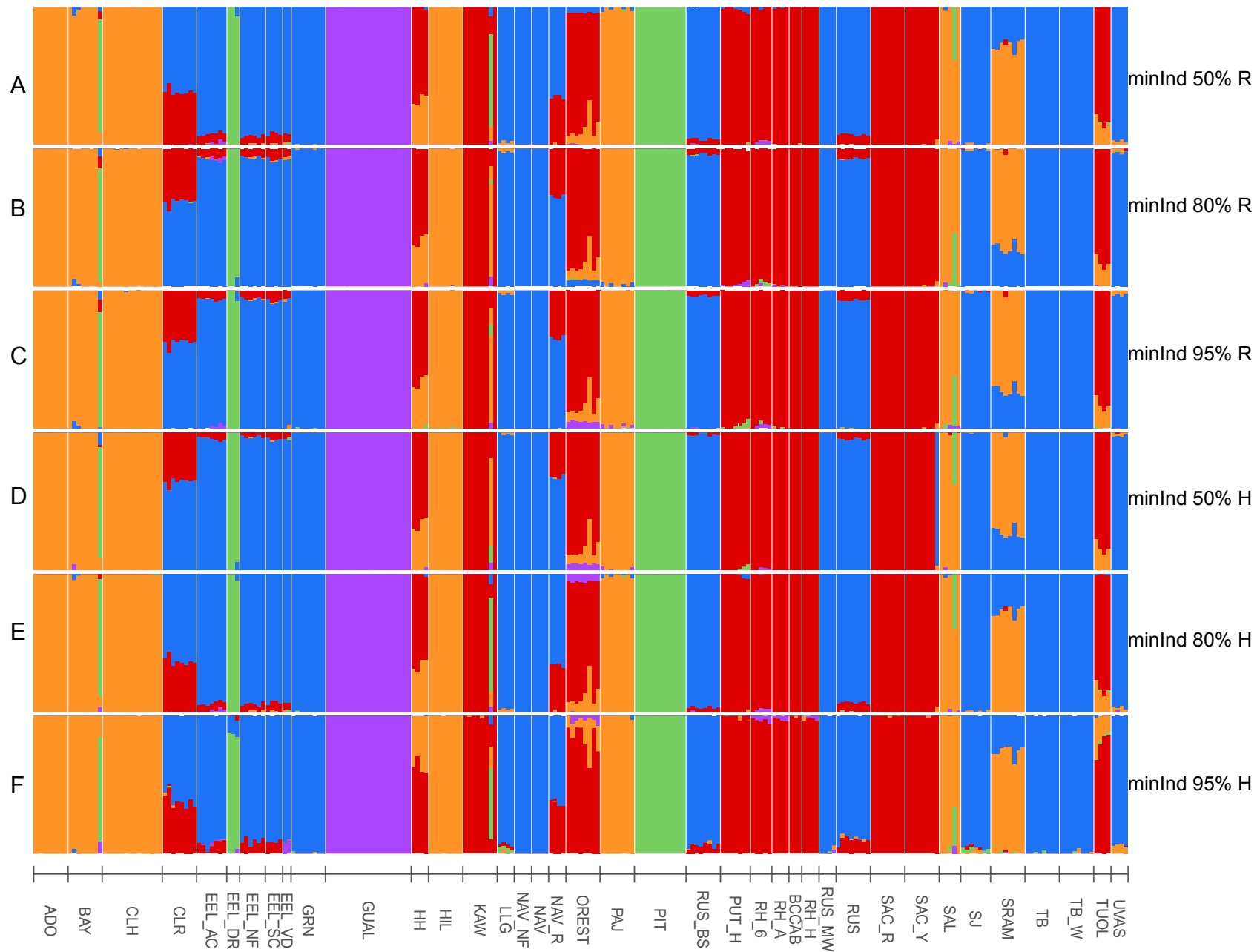

Supplement: S2 Fig — Loci present in at least 50%, 80% and 95% of individuals (minInd) based on original Pit Roach de novo assembly and a second filtering (50%, 80%, 95%) for a de novo assembly based on Hitch. (PDF) [file pone.0189417.s002.pdf]

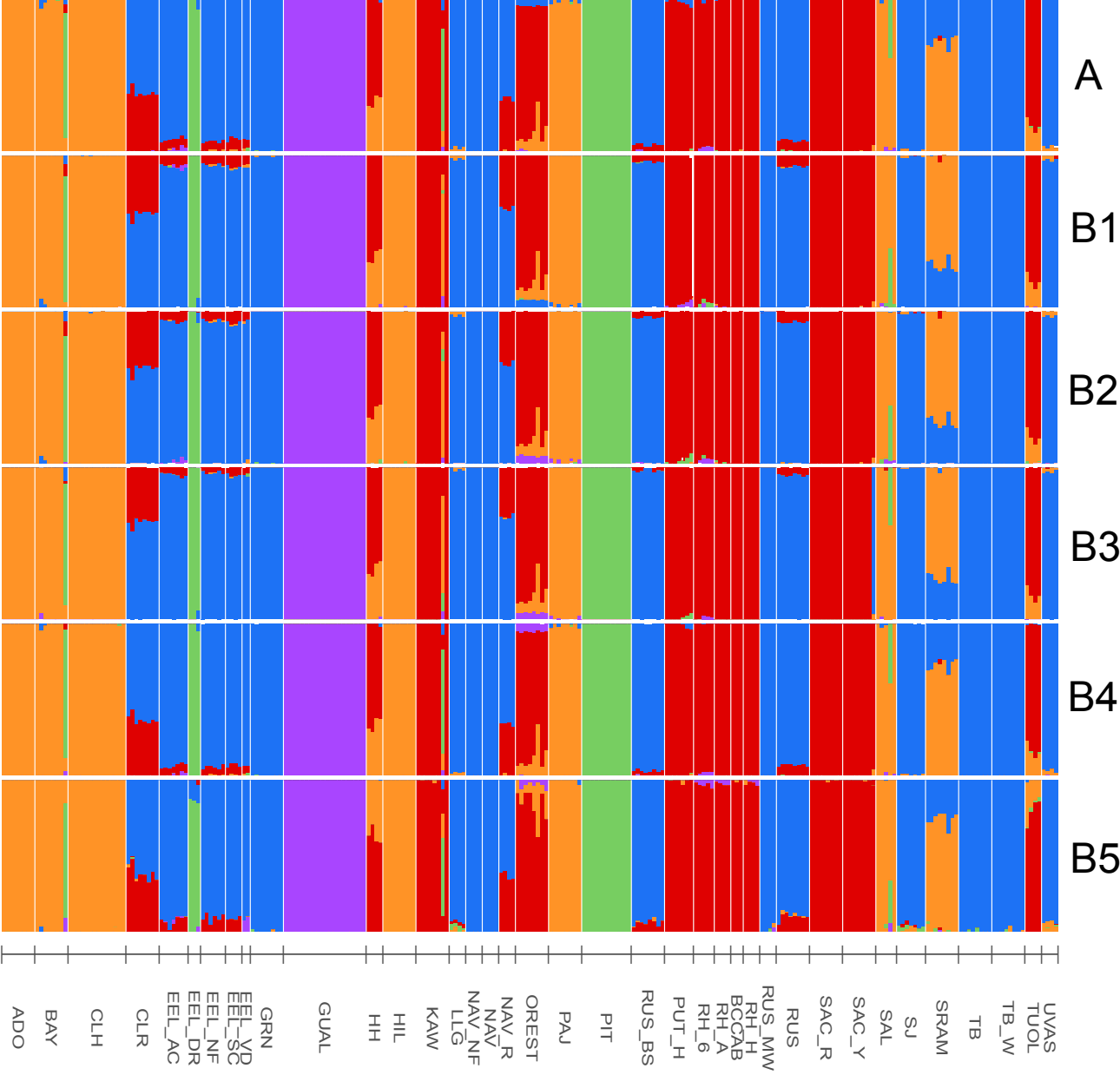

Supplement: S3 Fig — (PDF) [file pone.0189417.s003.pdf]

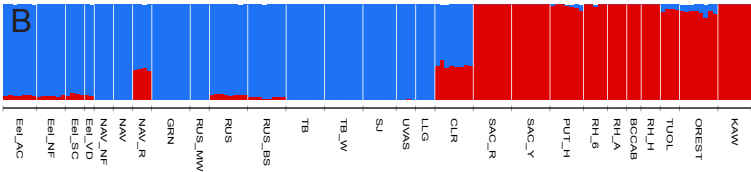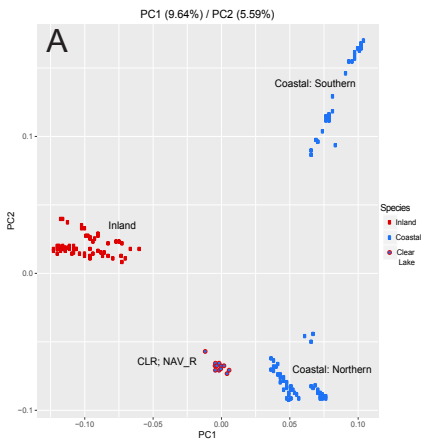

Supplement: S4 Fig — Analyses show individuals from the Clear Lake region and upper portion of the Navarro River (Rancheria) appear to be intermediate between proposed species. Colors are consistent across subfigures and nomenclature is consistent with abbreviations in S1 Table. (PDF) [file pone.0189417.s004.pdf]

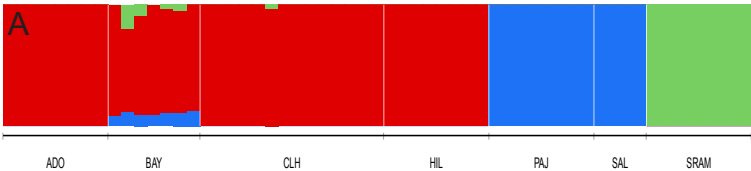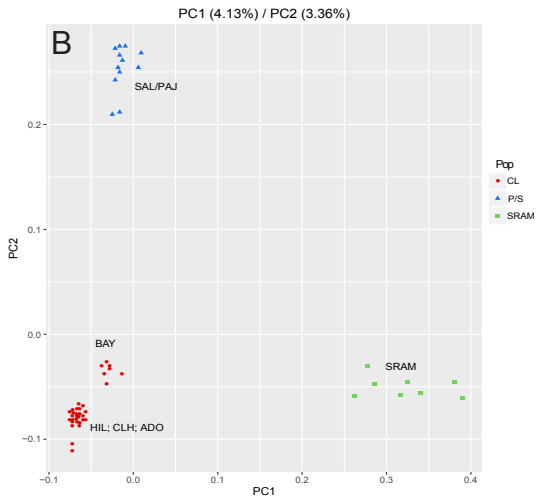

**C**

| $F_{st}$ | P/S   | SRAM  |
|----------|-------|-------|
| CL       | 0.072 | 0.133 |
| P/S      |       | 0.140 |

Supplement: S5 Fig — Substructure within samples shows three distinct populations, supported by FST values consistent with population structure seen throughout the study. Colors are consistent across subfigures and nomenclature is consistent with abbreviations in S1 Table. (PDF) [file pone.0189417.s005.pdf]
