# Supplementary material for: Genomics clarifies taxonomic boundaries in a difficult species complex
Source: PLoS One. 2017 Dec 12;12(12):e0189417. doi: 10.1371/journal.pone.0189417 (PMC5726641; doi:10.1371/journal.pone.0189417)
Supplement: S6 Fig — Colors are unique to each figure and do not correlate between figures. Results are identical to one SNP per locus used in paper. (PDF) [file pone.0189417.s006.pdf]

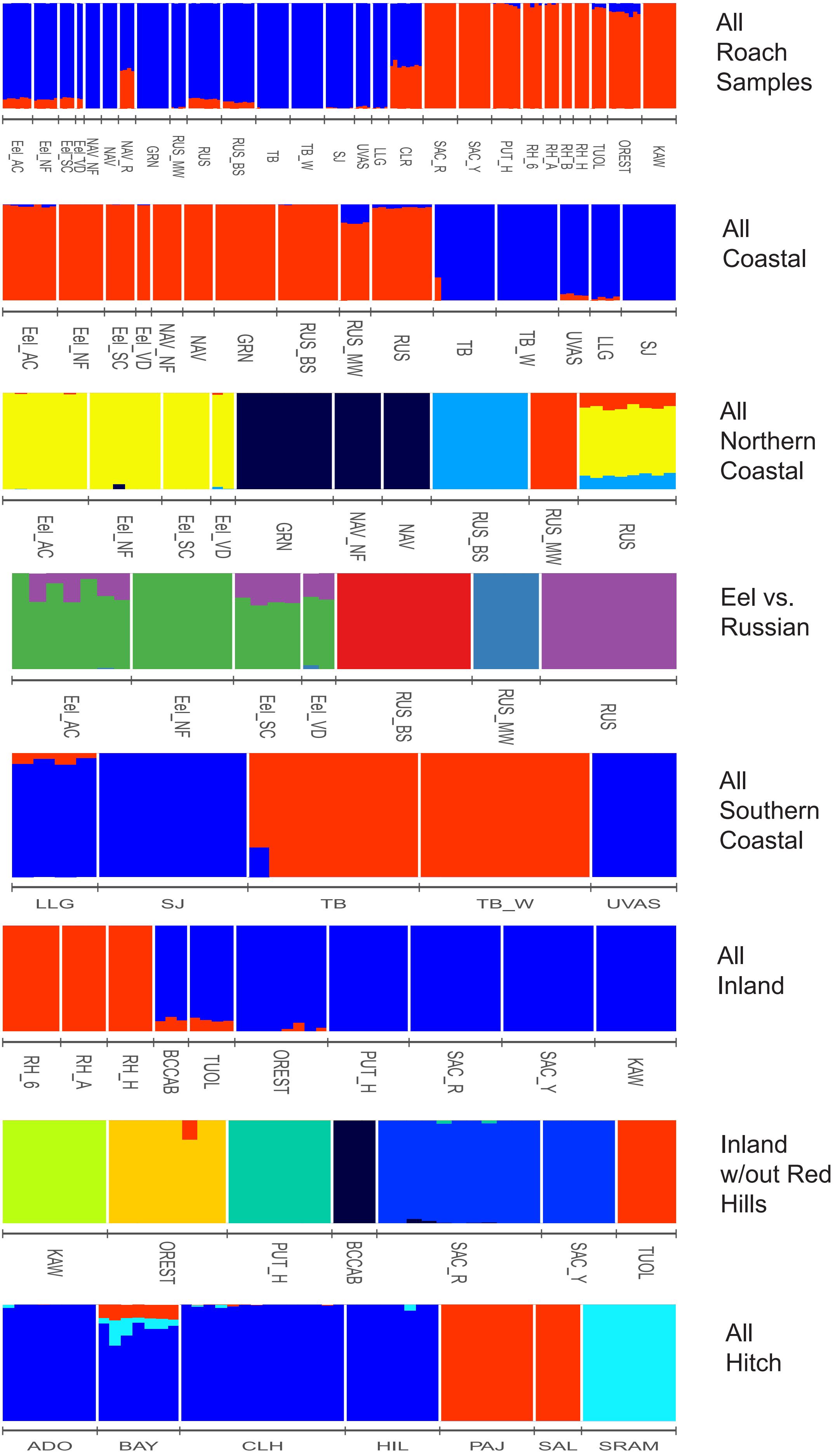

Eel_AC
Eel_NF
Eel_SC
Eel_VD
NAV_NF
NAV
NAV_R
GRN
RUS_MW
RUS
RUS_BS
TB
TB_W
SJ
UVAS
LLG
CLR
SAC_R
SAC_Y
PUT_H
RH_6
RH_A
RH_B
RH_H
TUOL
OREST
KAW
All Roach Samples
Eel_AC
Eel_NF
Eel_SC
Eel_VD
NAV_NF
NAV
GRN
RUS_BS
RUS_MW
RUS
TB
TB_W
UVAS
LLG
SJ
All Coastal
Eel_AC
Eel_NF
Eel_SC
Eel_VD
GRN
NAV_NF
NAV
RUS_BS
RUS_MW
RUS
All Northern Coastal
Eel_AC
Eel_NF
Eel_SC
Eel_VD
RUS_BS
RUS_MW
RUS
Eel vs. Russian
LLG
SJ
TB
TB_W
UVAS
All Southern Coastal
RH_6
RH_A
RH_H
BCCAB
TUOL
OREST
PUT_H
SAC_R
SAC_Y
KAW
All Inland
KAW
OREST
PUT_H
BCCAB
SAC_R
SAC_Y
TUOL
Inland w/out Red Hills
ADO
BAY
CLH
HIL
PAJ
SAL
SRAM
All Hitch
