# Supplementary material for: Genomics clarifies taxonomic boundaries in a difficult species complex
Source: PLoS One. 2017 Dec 12;12(12):e0189417. doi: 10.1371/journal.pone.0189417 (PMC5726641; doi:10.1371/journal.pone.0189417)
Supplement: S1 Table — (DOCX) [file pone.0189417.s007.docx]

| # | # sampled osampledIndiv | Code | Location | Latitude | Longitude |
| --- | --- | --- | --- | --- | --- |
| *Collected as Hitch* | | | | | |
| 1 | 8 | CL | Clear Lake | 38°57.607'N | -122°40.894'W |
| 2 | 6 | CLH | Clear Lake, CA | 39°02.153'N | -122°53.389'W |
| 3 | 8 | HIL | Hill Creek, Clear Lake, CA | 38°59.628'N | -122°51.694'W |
| 4 | 8 | ADO | Adobe Creek, Clear Lake, CA | 39°00.891'N | -122°52.251'W |
| 5 | 8 | BAY | San Francisco Bay, CA | 38°05.569'N | -121°43.311'W |
| 6 | 8 | SAL | Salinas River, CA | 36°33.353'N | -121°32.937'W |
| 7 | 8 | PAJ | Pajaro River, CA | 36°54.338'N | -121°40.613'W |
| *Collected as CA Roach* | | | | | |
| 1 | 4 | PT_DY | Dry Creek - Goose Lk - Pit R | 42°01.267'N | -120°38.767'W |
| 2 | 4 | PT_A | Ash Creek - Pit R | 41°09.654'N | -120°49.806'W |
| 3 | 6 | PIT | Pit River, CA | 41°16.461'N | -120°53.745'W |
| 4 | 8 | SAC_DY | Dye Crk - Sacramento R | 40°06.292'N | -122°02.351'W |
| 5 | 8 | SAC_DR | Deer Crk - Sacramento R | 39°58.209'N | -122°00.828'W |
| 6 | 8 | PUT_H | Hunting Crk - Putah Crk | 38°48.493'N | -122°22.530'W |
| 7 | 4 | TUOL | Woods Creek - Tuolumne | 37°57.751'N | -120°24.895'W |
| 8 | 4 | HH | Hetch Hetchy Reservoir | 37°57.056'N | -119°47.344'W |
| 9 | 4 | BCCAB | Becca B, Tuolumne R, CA | 37°47.882'N | -120°22.019'W |
| 10 | 4 | RH_H | Horton Crk, Tuolumne R, CA | 37°51.466'N | -120°27.404'W |
| 11 | 4 | RH_A | Red Hills – Andrew Crk - Tuolumne | 37°52.649'N | -120°30.682'W |
| 12 | 8 | RH_6 | Six Bit Gulch - Tuolumne | 37°51.413'N | -120°27.228'W |
| 13 | 8 | OREST | Orestimba Creek – Stanislaus | 37°17.416'N | -121°11.606'W |
| 14 | 8 | KAW | SF Kaweah R | 36°21.083'N | -118°46.713'W |
| 15 | 8 | CLK | Kelsey Crk - Clear Lake | 38°55.554'N | -122°50.675'W |
| 16 | 8 | HIL | Hill Creek, Clear Lake, CA | 38°59.628'N | -122°51.694'W |
| 17 | 8 | ADO | Adobe Creek, Clear Lake, CA | 39°00.891'N | -122°52.251'W |
| 18 | 8 | EEL_VD | Van Duzen R | 40°29.949'N | -123°58.575'W |
| 19 | 8 | EEL_NF | North Fork Eel R | 39°56.279'N | -123°20.970'W |
| 20 | 8 | EEL_SC | Scotia - Eel R | 40°28.471'N | -124°06.551'W |
| 21 | 8 | EEL_DR | Dos Rios - Eel R | 39°42.698'N | -123°20.942'W |
| 22 | 8 | EEL_AC | Angelo Coast - Eel R | 39°43.052'N | -123°39.227'W |
| 23 | 4 | NAV | Navarro R | 39°05.314'N | -123°29.339'W |
| 24 | 4 | NAV_NF | NF Navarro R | 39°09.393'N | -123°38.113'W |
| 25 | 4 | NAV_R | Rancheria Crk - Navarro R | 39°10.956'N | -122°54.711'W |
| 26 | 8 | GRN | Greenwood Crk - North Coast | 39°07.971'N | -123°42.263'W |
| 27 | 6 | NFG | North Fork Gualala River, CA | 38°45.773'N | -123°31.779'W |
| 28 | 6 | SFG | South Fork Gualala River, CA | 38°39.108'N | -123°21.872'W |
| 29 | 8 | GUAF | Fuller Crk - Gualala R | 38°41.490'N | -123°19.810'W |
| 30 | 8 | RUS | Russian R | 38°37.443'N | -122°50.514'W |
| 31 | 8 | RUS_BS | Big Sulfur Crk – Russian R | 38°47.188'N | -122°46.802'W |
| 32 | 4 | RUS_MW | Mark West Creek, Russian River, | 38°30.239'N | -122°44.859'W |
| 33 | 8 | TB_L | Lagunitas Crk, Tomales Bay, CA | 38°03.022'N | -122°45.593'W |
| 34 | 8 | TB_W | Walker Creek - Tomales Bay | 38°11.781'N | -122°50.921'W |
| 35 | 4 | UVAS | Uvas Creek - Pajaro R | 37°06.711'N | -121°45.261'W |
| 36 | 8 | GAT | Los Gatos Crk, San Jose, CA | 37°16.243'N | -121°56.947'W |
| 37 | 4 | LLEG | Llegas Creek - Pajaro R | 37°08.441'N | -121°47.138'W |
