# Supplementary material for: Genomics clarifies taxonomic boundaries in a difficult species complex
Source: PLoS One. 2017 Dec 12;12(12):e0189417. doi: 10.1371/journal.pone.0189417 (PMC5726641; doi:10.1371/journal.pone.0189417)
Supplement: S2 Table — All values were obtained with a minor allele frequency of 0.05, except those denoted by an asterisk. Clear Lake samples are not represented after inclusion at the All Samples and All Roach levels. (DOCX) [file pone.0189417.s008.docx]

| Hierarchical Level | # of Indiv | # of SNPs |
| --- | --- | --- |
| All samples | 255 | 690045* |
|  | 255 | 218087 |
| All Hitch | 57 | 155283 |
| All Roach | 198 | 163023 |
| Northern (Pit) | 12 | 63212 |
| Inland | 58 | 150768 |
| Inland minus Red Hills | 45 | 147177 |
| Gualala | 20 | 44153 |
| Coastal | 86 | 132953 |
| Coastal – Northern | 55 | 119847 |
| Coastal – Southern | 31 | 99640 |
| Eel /Russian | 39 | 113321 |
