# Supplementary material for: Genomics clarifies taxonomic boundaries in a difficult species complex
Source: PLoS One. 2017 Dec 12;12(12):e0189417. doi: 10.1371/journal.pone.0189417 (PMC5726641; doi:10.1371/journal.pone.0189417)
Supplement: S3 Table — (DOCX) [file pone.0189417.s009.docx]

| Cluster ID | Description | Tw/site | Tp/site |
| --- | --- | --- | --- |
| H | All hitch | 0.009446 | 0.007924 |
| H1 | Clear Lake | 0.008306 | 0.007697 |
| H2 | Pajaro/Salinas | 0.006789 | 0.007423 |
| H3 | San Ramon | 0.008233 | 0.009396 |
| P | Northern Roach | 0.005309 | 0.005625 |
| P1 | Ash Creek | 0.006120 | 0.006698 |
| P2 | Dry Creek, Pit R | 0.004291 | 0.005018 |
| P3 | Pit R | 0.003867 | 0.004712 |
| R | CA Roach | 0.013182 | 0.008881 |
| G | Gualala River | 0.002142 | 0.002687 |
| I | All Inland locations | 0.010445 | 0.008266 |
| I1 | All Red Hills locations (3) | 0.003249 | 0.003635 |
| I2 | All non-RH locations (7) | 0.009628 | 0.008017 |
| I2_BCCAB | Inland - Becca B Creek | 0.005521 | 0.006224 |
| I2_KAW | Inland - Kaweah R | 0.004242 | 0.004983 |
| I2_OREST | Inland - Orestima Creek | 0.006834 | 0.007335 |
| I2_PUTH | Inland - Putah Creek - Hunting | 0.006166 | 0.006600 |
| I2_SAC_R | Inland - Sacramento R – Deer Cr | 0.006201 | 0.006579 |
| I2_SAC_Y | Inland - Sacramento R – Dye Cr | 0.005946 | 0.006396 |
| I2_TUOL | Inland - Tuolumne R | 0.007601 | 0.007937 |
| C | All Coastal locations | 0.008111 | 0.007235 |
| CN | Coastal – Northern ssp. | 0.006304 | 0.006554 |
| CN1 | Coastal - Northern – NAV/GRN | 0.006331 | 0.006485 |
| CN2 | Coastal – Northern – TB/SJ | 0.003164 | 0.004193 |
| CS | Coastal – Southern ssp. | 0.005751 | 0.005592 |
| CS1 | Coastal – Southern - TB | 0.005962 | 0.006090 |
| CS2 | Coastal – Southern - SJ | 0.003145 | 0.003874 |
| RUS | Russian R | 0.006305 | 0.006526 |
| RUSBS | Russian R – Big Sulfur | 0.004592 | 0.005351 |
| RUSMW | Russian R – Mark West | 0.005565 | 0.006107 |
| EEL | All Eel locations (4) | 0.005415 | 0.006234 |
